# Effects of rare-earth light conversion film on the growth and fruit quality of sweet pepper in a solar greenhouse

**DOI:** 10.3389/fpls.2022.989271

**Published:** 2022-09-06

**Authors:** Yaxin Gao, Gongfeng Li, Bingbing Cai, Ziming Zhang, Ning Li, Yike Liu, Qingyun Li

**Affiliations:** College of Horticulture, Hebei Agricultural University, Baoding, China

**Keywords:** rare-earth light conversion film, sweet pepper, photosynthesis, growth, yield, quality

## Abstract

Light is an important environmental factor influencing plant growth and development. However, artificial light supplement is difficult to spread for its high energy consumption. In recent years, rare-earth light conversion film (RPO) covering is being focused on to be a new technology to study the mechanism of light affecting plant growth and development. Compared with the polyolefin film (PO), the RPO film advanced the temperature and light environment inside the greenhouse. Ultimately, improved growth and higher yield were detected because of a higher photosynthesis, Rubisco activity and Rubisco small subunit transcription. Compared with that in the greenhouse with polyolefin film, the plant height, stem diameter and internode length of sweet pepper treated with RPO increased by 11.05, 16.96 and 25.27%, respectively. In addition, Gibberellic acid 3 (GA3), Indole-3-acetic acid (IAA), Zeatin Riboside contents were increased by 11.95, 2.84 and 16.19%, respectively, compared with that with PO film. The fruit quality was improved, and the contents of ascorbic acid (Vc), soluble protein and soluble sugar were significantly higher than those of PO film, respectively, increased by 14.29, 47.10 and 67.69%. On the basis of improved fruit quality, the yield of RPO treatment increased by 20.34% compared with PO film. This study introduces an effective and low-energy method to study the mechanism and advancing plant growth in fruit vegetables production.

## Highlights

- In this study, we found an economical and effective method to enhance the plant growth and yield of sweet pepper in sola-greenhouse.

## Introduction

Light is essential for plant life and influences development, morphology, and metabolism. Upon absorption the photon of visible light, there will be an initial signal which can be transmitted through elaborate molecular and metabolic pathways and ultimately modifies the “behavior” of plants (Möglich et al., 2010). By perceiving light quantity and quality, plants get the information about whether light is present, the season and the day length, the direction of the light and the circadian clock changes through multiple receptors (de Wit et al., 2016). The photoreceptors are actually protein molecules. The photoreceptors or the light-harvesting complexes are always membrane located which is quite different with other chemoreceptors (Möglich et al., 2009). The light spectrum can be divided into several distinct sets including near-UVB (280-315 nm), ultraviolet light (300-400 nm), visible light (400-710 nm), and far-red light (710-760 nm; de Wit et al., 2016). There are different types of photoreceptors of plants to perceive different light spectrum with different wavelengths. Except the UVB receptor, all of the other photoreceptors contain a bound cofactor, known as the chromophore. It serves as the primary site of photon absorption and photon absorption of particular wavelengths actually takes place (Möglich et al., 2010). The larger is the chromophore, the longer is the wavelength it absorbs. The spectrum of photosynthesis is consistent with the absorption spectrum of chlorophyll, mainly concentrated in the red-orange and blue-violet regions. As we all know, light acts as an important developmental switch in morphogenesis, such adjustments in growth and development in response to light conditions are often established through changes in hormone levels, such as auxin (IAA), abscisic acid (ABA) and gibberellic acid (GA), and signaling (de Wit et al., 2016). Auxin is a key player in mediating the elongation phenotype. Light is an important environmental factor that can trigger many plant life processes, such as germination and morphogenesis. Germination starts with water uptake, which restart many cellular processes, including cell elongation. Cell elongation is the base for plant growth. In Arabidopsis seedlings, the main site of perception is in the cotyledons, however, in older Arabidopsis leaves, petiole elongation is induced by irradiation of the lamina through regulating the hormone contention and related genes expression (Tanaka et al., 2002; Kozuka et al., 2010). Red light may function as a positive factor for elongation and tomato fruit ripening, and in these processes, plant growth regulators, including GA, IAA, ABA and ethylene (ET) play a critical role in the regulatory mechanism (Sun, 2010; Liu et al., 2011; Zhang J. et al., 2020). However, continuous blue light decreases plant dry mass (Pham et al., 2019), plant height and root length (Wu et al., 2014) and delays shelf tomato fruits softening and ripening (Dhakal and Baek, 2014).

Light conversion film which is a kind of functional film is highly required to protect plants, foods and skin from UV radiation (Sirerol et al., 2015). It can convert the high-energy short waves (Ultraviolet light) in sunlight into low-energy long waves (Visible light) to enhance the photosynthetic efficiency of plants (Yang et al., 2020), thus showed a great potential in the field of horticulture. Most of the film use polyolefin or ethyl cellulose (EC) as the substrate and fluorescent materials as agent. At present, rare earth complexes were one of the most extensively studied fluorescent materials. Among them, the rare earth Eu complexes, which is the most often used as light conversion agent have a strong and pure red light (Zhang et al., 2022). The unsubstituted hydroxyl group can reacts with Eu 3+ to form a coordination bond, and sensitizes Eu 3+ to luminescence (Zhang Z. et al., 2020).

Sweet pepper (*Capsicum annuum* L.) is the main vegetable cultivated in greenhouses in China, and it has high requirements on light. However, weak light always occurs in solar-greenhouse during winters and springs, which inhibits growth and fruit development of sweet pepper (Sui et al., 2012). Therefore, exploring the mechanism and improving the greenhouse environment are necessary for enhancing sweet pepper growth during low-temperature seasons. Recent advances in control technologies, such as application of additional light source (light-emitting diode, LED), helps to ensure plant growth during the cultivation of greenhouse in low light seasons (Heuvelink et al., 2018). The rare-earth light conversion (RPO) film is practical, and cost-effective in solar greenhouse. The greenhouse film with a light transfer agent could considerably enhance tomato growth and fruit yield and quality by improving the light conditions in the solar greenhouse (Kang et al., 2022). Meanwhile, that RPO covering upon the greenhouse improved the air temperature and light intensity inside greenhouses, enhancing the heat preservation and light transmission rates (Garofalakis et al., 2006; Xin et al., 2019; Yu et al., 2019), and improved fruit quality and yield, such as tomato (*Lycopersicon esculentum* Miller; Xiao et al., 2022). At present, the application of RPO on sweet pepper is little reported and the mechanism is unknown.

Jizhong region in China is one of the main regions for cultivating sweet peppers, but the low temperature and weak light in the local area is not enough for sweet pepper growth, causing the low yield. Therefore, this study analyzed the effects of RPO film on the temperature and light environment in the solar greenhouse and the growth, photosynthesis, Rubisco activity and the relative transcription of Rubisco large/small subunit, content of endogenous hormone, fruit yield, and quality of sweet pepper, using the standard polyolefin (PO) film as a control. The results will provide a theoretical basis for in-depth exploring the application of RPO film in the production of fruit vegetable in low-temperature seasons.

## Materials and methods

### Seedlings and solar-greenhouse

**Figure 1:**
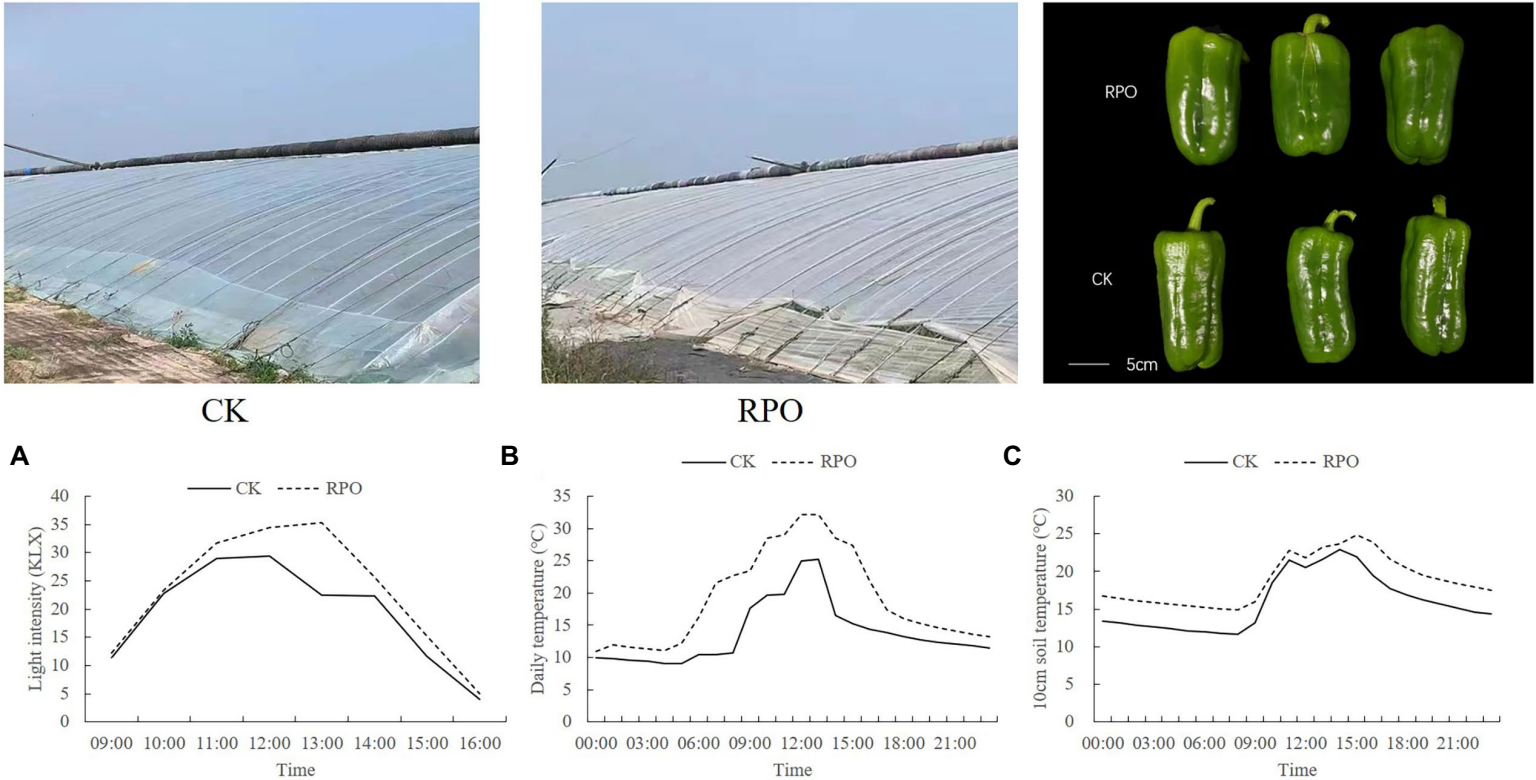
Effects of RPO film on the changes of sunlight intensity and temperature in the greenhouse. **(A)** Daily variation of light intensity; **(B)** air temperature and **(C)** soil (10 cm soil layer) temperature.

Sweet pepper seedlings were grown in a thick soil wall solar greenhouse of the Hebei Kangcheng Modern Agricultural Product Development Co., Ltd. (Shixiang Village, Baoding City, Hebei Province; 115.66° E, 39.16° N) from August 2021 to January 2022. The greenhouse was 100 m long and 5.4 m high, with an inner span of 11.5 m. Two kinds of films were used to cover on the greenhouses, including RPO (Rare earth Eu agent was added into Polyolefin film; Inner Mongolia Sunming Co., Ltd., 0.1 mm thickness) as the treatment and Polyolefin film (PO, 0.1 mm thickness) film as the control (CK), as shown in Figure 1. At least 100 m long and 10 m wide films were prepared, followed by covering on the greenhouse steel. In the sequence of top, two terminals (east and west) and front end to fix the films. The two greenhouses were both covered with thatch which was made of rice straw to keep warm in low-temperature seasons. The seedlings were grown in the soil in the greenhouse mulched with these two kinds of films, respectively, with a large row spacing of 80 cm, a small row spacing of 40 cm, and a plant spacing of 35 cm and irrigated in furrows. The planting area is 1,100 m2, and each greenhouse has about 83 rows. Water and fertilizer management, pest and disease control were carried out following the conventional practices. The sweet pepper plants that with six true leaves were planted on 25 August 2021 and started harvesting on 20 November 2021, and these sweet pepper plants were pulled out on 10 January 2022.

### Measurement of light intensity, spectrum and temperature

Light intensity, air temperature, and soil temperature were continuously monitored from October 2021 to January 2022. The spectrum inside the greenhouse were measured, and the spectral ratios were calculated every 30 days from 1 November 2021. The light intensities inside and outside the greenhouse were measured, and the light transmittance was calculated every 30 days. Plant growth indicators, functional leaf anatomy, chlorophyll content, photosynthetic parameters, ribulose-1,5-bisphosphate carboxylase/oxygenase (Rubisco) activity, and Rubisco gene expression were measured 90 days after planting (25 November, early harvest). The commercial fruits’ endogenous hormone content and other quality indicators were measured at harvest stage, and the fresh and dry weights and root activity were measured at 135 days after planting (uprooting day).

The spectrum of the greenhouse film was measured using the QE65000 spectrometer (Ocean Optics, Shanghai, China) in the middle of the greenhouse. At 3 m distance from the film and 1.5 m height from the ground, between 09:00 and 11:00. The light intensity that used for calculating the transmittance was measured using an Item #3415F Quantum light meters (Spectrum Technologies, Inc., United States) installed 1.5 m above the ground in the middle of the greenhouse. The value of light intensity was the average of three measurements. The transmittance (T) was calculated as follows: T = Ri/R0 × 100%, where Ri and R0 are the light intensity parallel to the greenhouse film inside and outside the greenhouse, respectively. The light intensity was measured simultaneously as that of the transmission spectrum of the greenhouse film.

The inner environment including air temperature and light intensity were recorded using the Smart Data Logger (Hangzhou Dlog Tech, Hangzhou, China) that installed in the middle of the greenhouse and above the ground of 1.5 m. The ET60 moisture meter (Oriental Intelligence Technology Co., Ltd. China) was used to record the soil temperature at 10 cm soil depth.

### Measurement of morphological parameters

Plant height, stem diameter, leaf length, leaf width, and internode length were measured using a ruler and a vernier caliper. The stem diameter was determined after planting 90 days. The length between the 2nd and 3rd nodes counted from the plant base was measured as the internode length. Leaf length and leaf width of the second true leaf from the bottom of the plant which were fully developed were measured. 15 individual plants were selected randomly for the measurements.

The sweet pepper plants were cleaned with distilled water and the surface moisture was air-dried. The fresh weights of the aerial part and the underground part were measured using an electronic balance with an accuracy of 0.01 g. Then each sample in an envelope was put into an oven at 105°C for 30 min followed by drying at 70°C until the dry weight was constant. The dried samples were used for the measurement of dry weight. Root activity is representation of absorbing ability of root and was determined using 2, 3, 5-Tripheyl-2H-tetrazolium chloride (TTC) method (Li, 2000). Each measurement was repeated five times.

### Optical electron microscope

Leaf cell structure was analyzed following the method by Vitha and Osteryoung (2011). The fourth fully expanded leaf from the top of a plant was selected, and the middle of the leaf (0.5 cm × l cm) was cut with a razor blade. The leaf blade was then placed in a vials bottle containing 4 ml of 70% standard fixative (FAA), vacuumed, and stored at 4°C. Leaves were made into thin section. These leaf sections were double-stained with saffron green and observed under an OLYMPUS BX51M microscope (Beijing, China).

Meanwhile, clear nail polish (Yiwu Meidianyuan Cosmetics Co., Ltd. Zhejiang. China) was applied in the middle of the fourth fully expanded leaf from the top and allowed to dry completely. Then, the varnish was removed using tweezers, and the adhering epidermal tissue was observed under the OLYMPUS microscope to measure the stomatal length.

### Photosynthesis and Rubisco activity

The fourth fully expanded, which were the most functional leaves from the top were selected to determine the net photosynthetic rate (Pn) using the YZQ-100A portable dynamic photosynthesis meter (YI ZONG QI TECHNOLOGT CO., LTD. Beijing, China), with the light quantum flux density (PFD) set at 1000 μmol (m^-2^ s^-1^). The chlorophyll content and Rubisco activity of the same leaves were determined after the net photosynthetic rate was measured. The chlorophyll content was measured as described by Li (2000). Rubisco activity was measured using the Rubisco enzyme immunoassay kit (MM-62512O2, Jiangsu Meimian industrial Co., Ltd., China) by Beijing ZKGX Research Institute of Chemical Technology (Beijing, China). Each measurement was repeated five times.

### RNA isolation and quantitative real-time PCR

The same leaf that used for the measurement of Rubisco activity was quick frozen in liquid nitrogen, and used for the determination of gene expression level of Rubisco large subunit (*RbcL*, KT779528) and Rubisco small subunit (*RbcS*, KC176707.1). Total RNA was isolated using the Eastep^®^ Super Total RNA Extraction Kit (Promega, LS1040, Beijing, China) according to the manufacturers’ instructions and diluted to one uniform concentration. The first strand cDNA was synthesized from 1 μg total RNA with the PrimeScript^TM^ RT reagent Kit (Perfect Real Time; TaKaRa, Bio Inc., China) according to the manufacturers’ instructions. The quantitative real-time PCR was used to analysis the expression patterns of photosynthesis-related genes *RbcS* and *RbcL* on a CFX Touch Real-time PCR system (Bio-Rad Laboratories, Hercules, CA, United States). Ubi3 (AY486137.1) was used as the internal reference gene. The PCR primers were designed using Primer Premier 5 software to avoid the conserved regions and to amplify products of 150-300 bp. Primer sequences are shown in detail in Supplementary Table S1. The real-time PCR was performed using the SYBR R Premix Ex Taq^™^ II (TaKaRa, Bio Inc., China), and the analysis of each type of sample was repeated five times as technical repeats. The analysis of relative mRNA expression data was performed using the 2^-ΔΔCt^ method (Wan et al., 2011).

### Measurement of endogenous hormone content in fruit

Sweet pepper fruits of uniform size and maturity were selected at the peak of the fruiting period, quick frozen in liquid nitrogen, and used for the determination of the content of GA3, ABA, IAA, and ZR using enzyme-linked immunosorbent assay (ELISA) kit (Chinese Agriculture University, Beijing, China; Wang et al., 2015) according to the manufacturers’ instructions.

### Measurement of soluble protein, ascorbic acid, titratable acids and free amino acids

The content of soluble protein of the fruit was performed using the Coomassie Brilliant Blue G-250 staining method (Li, 2000). The content of ascorbic acid (Vc) was carried out using the 2, 6-dichlorophenol indophenol titration method (Li, 2000). The content of titratable acids was performed using the sodium hydroxide titration method (Li, 2000), and the content of free amino acids was carried out using the ninhydrin colorimetric method (Li, 2000). Every treatment was repeated three times, and 10 plants were randomly selected for each replicate.

### Measurement of yield

The yield was recorded during the whole harvesting stage (from the start of the harvest to the pulling period). Every treatment was repeated five times and 15 plants were randomly selected for each replicate.

### Data analysis

Differences between groups were tested using Student’s t-test in Statistical Product and Service Solutions, version 26.0 (IBM, New York, United States), and data are presented as the mean ± one standard deviation (SD). Lower letters indicate significant differences between two groups according to t-test (*p* < 0.05).

## Results

### Effect of RPO film on spectral distribution and light transmission rate

The spectral distribution of radiations in the greenhouse is shown in Table 1. The analysis of the transmittance spectra showed that the RPO film projected 25.72, 13.29, and 6.73% less UV, violet, and green light bands and 8.03, 7.00, and 6.57% more blue, red-orange, and far-red light than CK (*p* < 0.05). The light transmission rate of the RPO film was 11.33% higher than that of the CK. These results indicated that RPO increased the blue, red-orange, far-red light and transmittance, but decreased UV, purple light and green light in the greenhouse.

**Table 1:** Effect of RPO film on the spectral transmission ratio.

Treatment	UV-A (%) (300–400 nm)	Purple light (%) (400–440 nm)	Blue light (%) (440–510 nm)	Green light (%) (510–610 nm)	Red orange light (%) (610–710 nm)	Far red light (%) (710–760 nm)	Transmittance /%
CK	8.32 ± 0.17a	5.87 ± 0.17a	15.31 ± 0.23b	30.18 ± 0.25a	27.56 ± 0.31b	11.41 ± 0.20b	79.64 ± 0.60b
RPO	6.18 ± 0.01b	5.09 ± 0.17b	16.54 ± 0.29a	28.15 ± 0.68b	29.49 ± 0.35a	12.16 ± 0.12a	88.66 ± 1.49a

All values are presented as the mean ± SD (*n* = 5). Lowercase letters indicate that the mean values are significantly different among samples (*p* < 0.05).

### Effect of RPO film on daily variations in light intensity and temperature in a greenhouse

As the results shown in Figure 1A, the light intensity in the greenhouse with RPO film and CK (PO film) increased in the morning (9:00-13:00, the thatch was removed at 9:00 am) and then decreased in the afternoon (13:00-16:00, the thatch was covered again at 16:00 pm). The light intensity in the greenhouse with RPO film was higher than CK and reached the highest at 13:00, when the difference between RPO and CK was the maximum (17.67%). Meanwhile, the changes of the temperature showed a similar trend with light intensity in RPO and CK greenhouses (Figure 1B). From 0:00 to 21:00, the temperature in the greenhouse with the RPO film was always higher than that of CK, with the highest temperature at 13:00, 29.32% higher than CK (*p* < 0.05).

The changes observed in soil temperature (10 cm soil layer) changed in a trend of bimodal curve; the soil temperature of CK and RPO treatments reached the highest at 14:00, and the temperature of the soil in the greenhouse with RPO was 12.26% (24.63°C) higher than that of CK (22.88°C; Figure 1C).

### Effect of RPO film on the tissue structure of leaves

**Figure 2:**
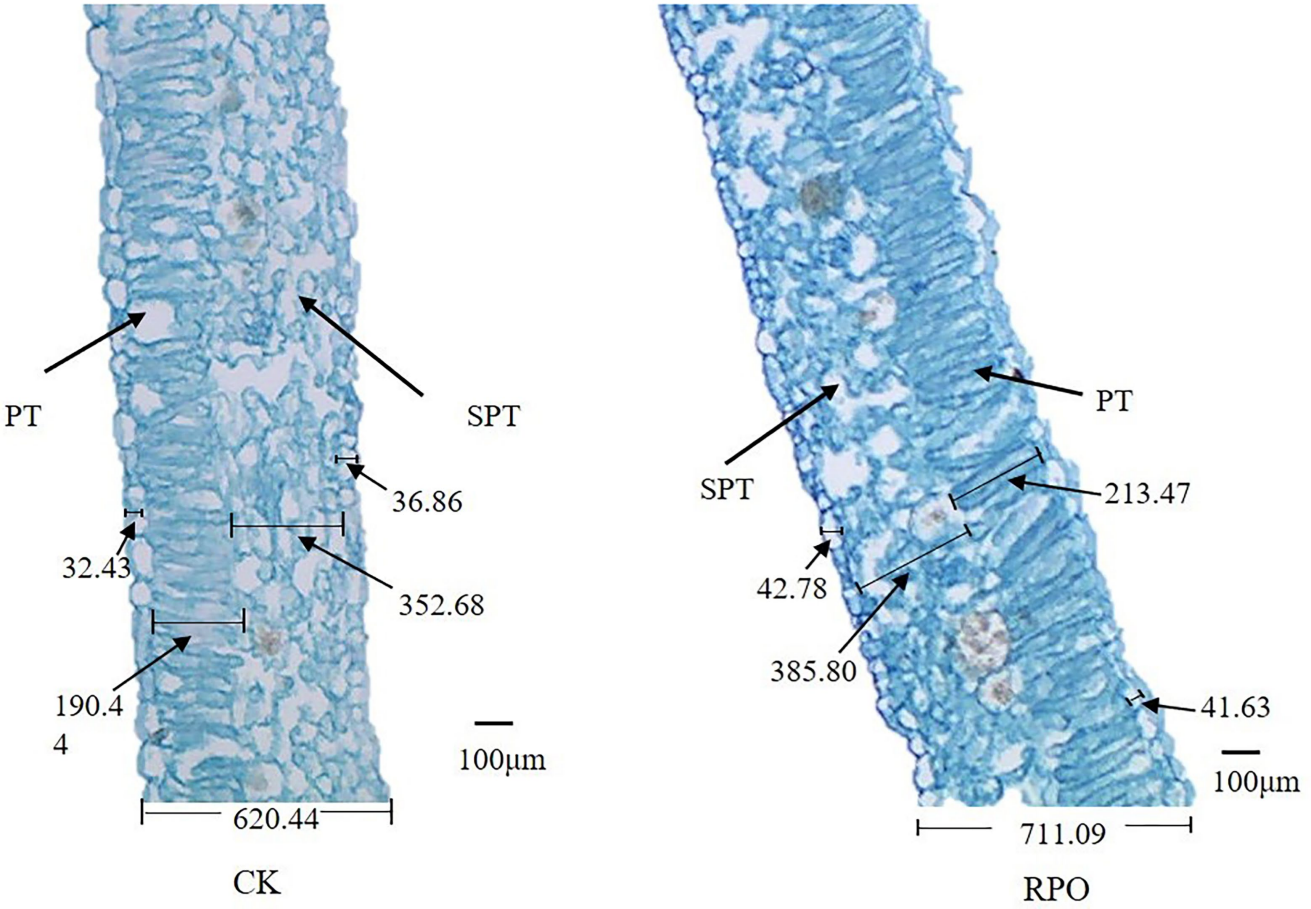
Effect of RPO film on the structure of sweet pepper functional leaves. SPT: spongy tissue; PT: palisade tissue.

The present study showed that RPO influenced the leaf structure of sweet pepper plants sampled at 90 days after planting (Figure 2). The palisade tissue cells near the proximal axis were long and columnar and neatly and closely arranged in the cross-section samples. Compared with CK, the leaf palisade tissues of sweet pepper plants grown in the greenhouse with RPO film were closely arranged, while the spongy tissue cells were irregular in shape, round, loosely arranged, and had significant gaps.

To observe whether RPO affects the tissue structure of leaves, we further analyzed the stomatal length of pepper leaves (90 days after planting). As shown in Table 2, the leaves under RPO film were significantly thicker than that of CK (14.61%), the thickness of upper and lower epidermis were significantly higher than that of CK (31.91, 12.94%), and the length of palisade tissue, spongy tissue, and stomata was significantly longer than that of CK (12.09, 9.39%).

**Table 2:** Effect of RPO film on the structure of mature sweet pepper leaves.

Treatment	leaf thickness/ um	Upper epidermal/um	Lower epidermal/um	Palisade parenchyma cells/um	Spongy parenchyma cells /um	stomatal length/um
CK	620.44 ± 1.62b	32.43 ± 2.24b	36.86 ± 0.35b	190.44 ± 5.51b	352.68 ± 9.03b	85.40 ± 2.70b
RPO	711.09 ± 2.91a	42.78 ± 2.32a	41.63 ± 1.29a	213.47 ± 3.79a	385.80 ± 3.94a	108.20 ± 2.39a

All values are presented as the mean ± SD (*n* = 5). Lowercase letters indicate that the mean values are significantly different among samples (*p* < 0.05).

### Effects of RPO film on fruit endogenous hormone content

Plant hormones, especially IAA, GA3, ZR, and ABA, play important roles in regulating fruit growth and development. In this paper, in order to explore the effect of RPO film on the endogenous hormones, the second fruits growing on the plants were sampled and the contents of GA3, IAA, ABA and ZR were measured. As the results shown in Table 3, contents of GA3, IAA, and ZR of sweet pepper fruits grown under RPO film were significantly higher than CK (11.95, 2.84, and 16.19%, respectively); however, the ABA content was significantly lower in the fruits under RPO (9.26%).

**Table 3:** Effect of RPO film on endogenous hormones in sweet pepper fruits.

Treatment	GA3 (ng g^-1^ FW)	ABA (ng g^-1^ FW)	IAA (ng g^-1^ FW)	ZR (ng g^-1^ FW)
CK	3.18 ± 0.01b	69.58 ± 0.15a	19.37 ± 0.02b	3.15 ± 0.01b
RPO	3.56 ± 0.02a	63.14 ± 1.44b	19.92 ± 0.07a	3.66 ± 0.02a

All values are presented as the mean ± SD (*n* = 5). Lowercase letters indicate that the mean values are significantly different among samples (*p* < 0.05).

### Effect of RPO film on photosynthesis and Rubisco activity of sweet pepper leaves

**Figure 3:**
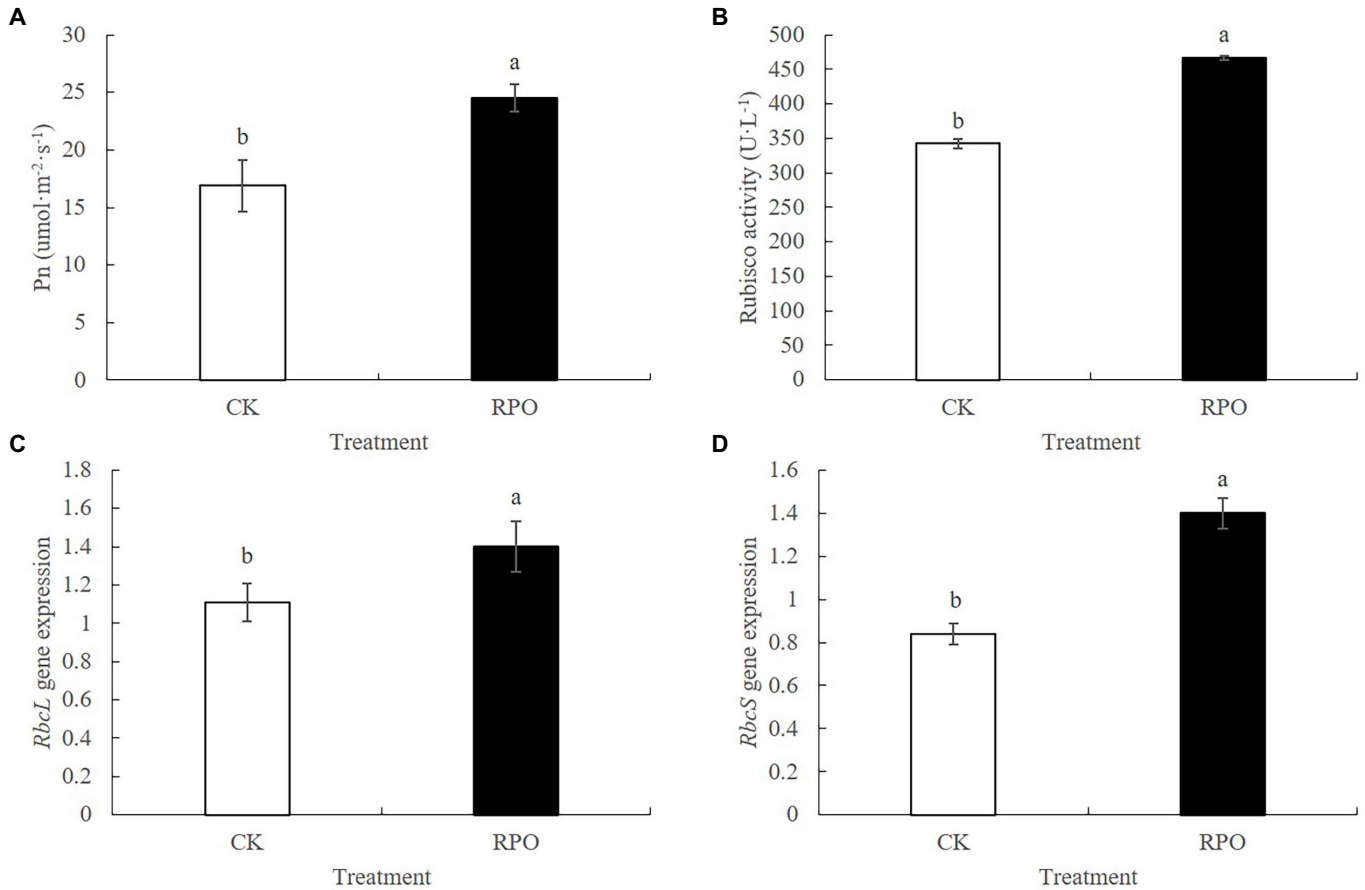
Effect of RPO film on Photosynthetic Correlation Index of sweet pepper leaves. **(A)** Photosynthetic rate; **(B)** Rubisco activity; **(C)** *RbcL* gene expression; **(D)** *RbcS* gene expression.

To study the effect of RPO film on the photosynthesis, the contents of chlorophyll and carotenoids were detected. As shown in Supplementary Table S2, the contents of chlorophyll a and b under RPO film were 17.75 and 28.49% higher than that of CK (*p* < 0.05) sampled at 90 days after planting, respectively. Also, higher carotenoids content was detected in the plants under RPO film, but there was no significant difference with that of CK. The net photosynthetic rate (Pn) was also determined. Higher Pn was indeed detected in the plants growing in the greenhouse with RPO film (Figure 3A). In addition, the Rubisco activity of plants under RPO film was significantly higher than that of CK (Figure 3B). The Pn and Rubisco activity of plants under RPO film was 45.32 and 36.14% higher than that of CK, respectively.

### Effect of RPO film on the transcription of *RbcL* and *RbcS*

The expression level of *RbcS* was significantly increased by 66.67% with RPO film coverage compared with CK. Meanwhile, the expression level of *RbcL* was also increased but no significant difference was observed between the plants under RPO film and CK (Figures 3C,D).

### Effect of RPO film on the growth of sweet pepper plants

As shown in Supplementary Table S3, the height, stem diameters, and internode length of sweet pepper plants grown with RPO were 11.05, 16.98, and 25.27% (*p* < 0.05) higher than those of CK, respectively. Meanwhile, leaf length and leaf width of plants of RPO film were 1.70 and 4.21% higher than those of CK, respectively, but no significant differences were detected (*p* > 0.05). These observations indicated that the RPO film promoted the elongation and thickening of sweet pepper stems.

In addition, both shoot dry and fresh weights with RPO film were significantly higher than that of CK (Supplementary Table S4). The fresh root weight of plants grown with RPO film was 36.84% higher than that of CK. Meanwhile, stem, leaf, and root dry weights increased by 61.57, 34.02, and 36.33% when grown under RPO film compared with CK. In addition, significantly higher root activity (30.18%) of plants with RPO film was detected than that of CK (Supplementary Figure S1).

### Effect of RPO film on the fruit quality and yield

When covered by RPO film, the fruit length, fruit width, average fruit weight and yield per unit area were increased by 13.80, 6.80, 30.49 and 20.34%, respectively (Table 4).The contents of Vc, soluble protein, and soluble sugar of sweet pepper fruits in the greenhouse with RPO film increased by 14.29, 47.10, and 67.69%, respectively, compared with CK (*p* < 0.05). The organic acid of fruits in the greenhouse with RPO film was significantly reduced compared with CK (23.81%; *p* < 0.05). However, no significant difference was observed in the content of free amino acid (Table 5).

**Table 4:** Effect of RPO film on fruit yield of sweet pepper.

Treatment	Fruit length (mm)	Fruit width (mm)	Number of fruits per plant	Average weight per fruit (g)	Yield (kg·hm-2)
CK	114.69 ± 7.42b	53.55 ± 3.74b	5.90 ± 0.10a	135.16 ± 3.27b	18259.09 ± 18.96b
RPO	130.52 ± 12.91a	57.19 ± 2.21a	5.90 ± 0.10a	176.37 ± 2.06a	21972.73 ± 17.25a

All values are presented as the mean ± SD (*n* = 5). Lowercase letters indicate that the mean values are significantly different among samples (*p* < 0.05).

**Table 5:** Fruit quality of sweet pepper fruits grown under RPO and PO film.

Treatment	Vc (mg·g-1)	Soluble protein (mg·g-1)	Organic acid (%)	Free amino acids (mg·g-1)	Soluble sugars (g·per 100 g)
CK	0.35 ± 0.001b	1.55 ± 0.05b	1.68 ± 0.14a	0.75 ± 0.06a	1.30 ± 0.04b
RPO	0.40 ± 0.001a	2.28 ± 0.11a	1.28 ± 0.14b	0.77 ± 0.08a	2.18 ± 0.03a

All values are presented as the mean ± SD (*n* = 5). Lowercase letters indicate that the mean values are significantly different among samples (*p* < 0.05).

### Correlation analysis of RPO on sweet pepper quality and yield

It can be seen from Supplementary Table S5 that the Vc content of sweet peppers was significantly correlated with soluble sugar (*R* = 0.59), average fruit weight (*R* = 0.671) and yield (*R* = 0.645); soluble protein was significantly negatively correlated with organic acids (*R* = −0.831); extremely significantly correlated with soluble sugar (*R* = 0.983), average fruit weight (*R* = 0.974) and yield (*R* = 0.976), and significantly correlated with fruit length (*R* = 0.743); Acid was significantly negatively correlated with soluble sugar (*R* = −0.839), average fruit weight (*R* = −0.820) and yield (*R* = −0.851); soluble sugar was extremely significantly correlated with average fruit weight (*R* = 0.990) and yield (*R* = 0.997), and significantly correlated with fruit length (*R* = 0.670); fruit length was significantly correlated with yield (*R* = 0.644); average fruit weight was significantly correlated with yield (*R* = 0.993). very significantly correlated.

### Principal component analysis (PCA) on yield and quality of sweet pepper by RPO

**Figure 4:**
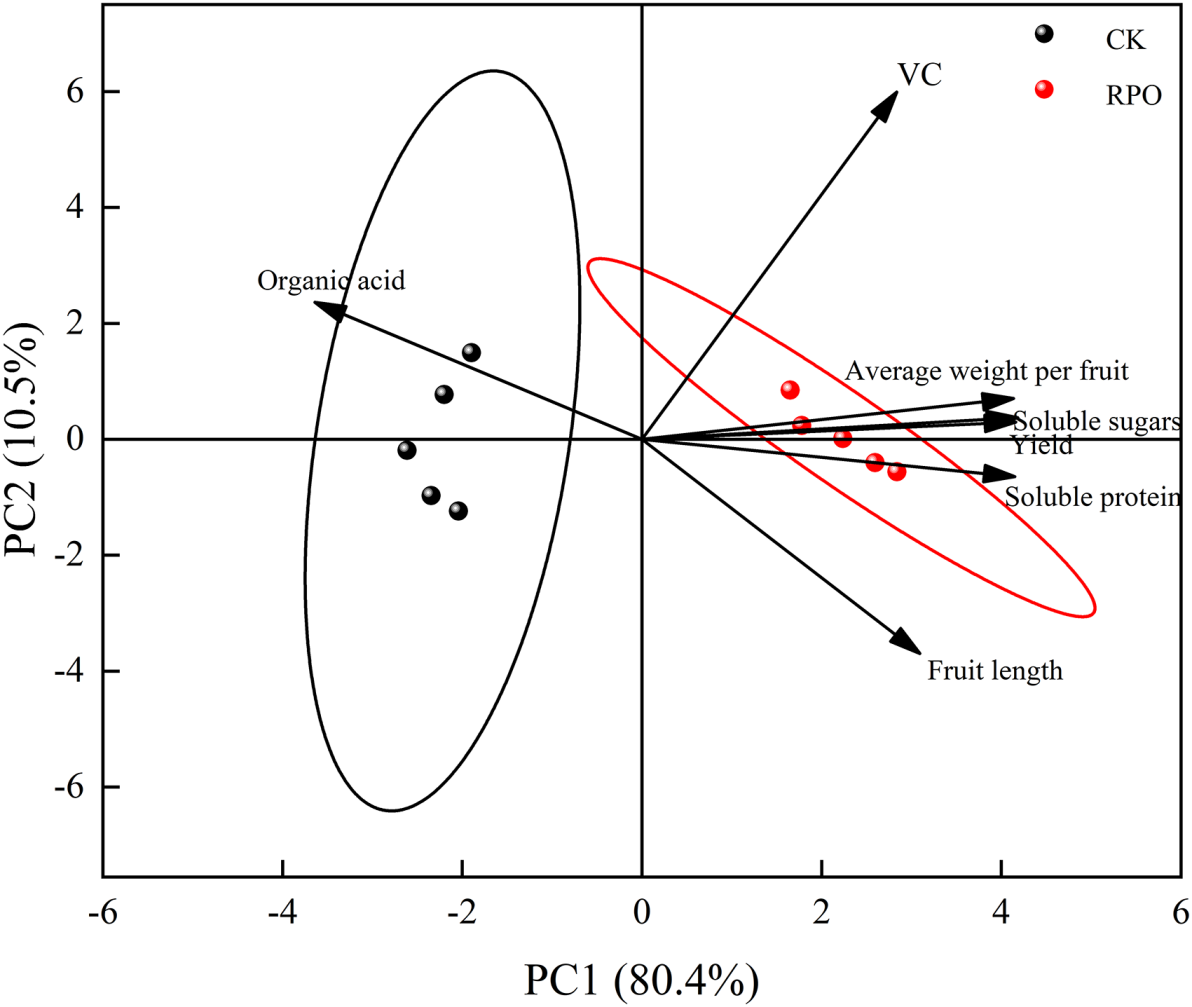
Principal component analysis results of RPO on sweet pepper quality and yield.

The quality and yield of sweet peppers will be subject to principal component analysis under different shed films. The results are shown in Figure 4. The first principal component explained 80.4% of the variance variation and the second principal component explained 10.5% of the variance. In terms of principal component analysis, the quality and yield responses to RPO film were clearly distinct from the responses to the PO film (CK). There was a positive correlation between organic acid and principal component 1, and a positive correlation between fruit length and soluble protein. While Vc, average fruit weight, soluble sugar and yield contributed more to the principal component 2. The factor scores showed that the principal component 1 could almost distinguish the effects of the RPO on the quality and yield of sweet pepper.

## Discussion

Together with temperature, moisture, oxygen, and nutrients, light is another important environmental factor that can influence morphological establishment, especially in sun-loving species (de Wit et al., 2016). Different light signals can trigger changes in plant growth and development, and these changes are typically regulated by plant hormones (de Wit et al., 2016). The study on lettuce (*Lactuca sativa* L.cv.) found that photosynthetic indices and nutritive value were response to constant or parabolic blue and/or red light (Borthwick et al., 1952; Samuoliene et al., 2020). The main photoreceptor of red light is phytochrome B (phyB) and the main photoreceptor of far-red light is phytochrome A (phyA; Shinomura et al., 1996; Guo et al., 2021), in which the ABA signal was involved (Lee et al., 2012). Our study found that phy A and phy B can be activated by red light and lead to GA3 biosynthesis (Tables 1, 3). Phy B can be inactivated by a pulse of far red light and leads to the biosynthesis of ABA in endosperm (de Wit et al., 2016). Different result was obtained in this paper probably due to the red-orange light and far-red light which were both strengthened in the greenhouse covered by RPO film (Table 1) and the far-red light in the greenhouse had lasted long.

Plant photomorphogenesis was induced by light perception. Light perception for.

phytochrome-mediated photomorphogenesis predominantly takes place in the mesophyll (Endo et al., 2005; Warnasooriya and Montgomery, 2009). The photoreceptors involved and the following rapid changes in development underline the significance of light perception for seedling establishment (de Wit et al., 2016). The activated photoreceptors can transduce the photomorphogenesis signal. Together with the suppressor of phytochrome A (SPA) proteins, the repressors constitutive photomorphogenic 1 (COP1) forms an E3 ubiquitin ligase complex, COP1-SPA complex. COP1-SPA can be inactivated by light signaling through the phytochromes and the cryptochromes, and result in the accumulation of positive regulators of photomorphogenesis, including elongated hypocotyl 5 (HY5; Huang et al., 2014; Lu et al., 2015; Sheerin et al., 2015). Among the numerous targets of HY5 are many regulators of hormone signaling, including ABA, GA, auxin and so on (Lau and Deng, 2010; Wang et al., 2012; Oh et al., 2014). Studies have demonstrated that purple light induces IAA oxidase activity, resulting in reduced IAA content in wheat. While treated with blue light for 45 days, the concentration of IAA in the stem of strawberry seedlings was significantly higher than that treated with other lights (Yonghua et al., 2005), consistent with the present study (Table 3), and that is why a higher plant height and a larger internode length was determined in this study (Supplementary Table S3).

Photosynthesis is the basis of almost all life on earth, and it is the main component of crop yield that contributes to the source such as photosynthates (Nutan et al., 2020). It can be greatly influenced by light intensity and the photosynthates acts as the basis for plant growth and yield formation. In the pathway of photosynthesis, RuBP carboxylase/oxygenase (EC 4.1.1.39, Rubisco) catalysis the reaction of binding of CO2 to the acceptor-molecule ribulose-1,5-bisphosphate (RuBP) to form two molecules of 3-phosphoglycerate, and is one of the limiting enzymes in the Calvin cycle (Spreitzer and Salvucci, 2002). The character of catalyzing both carboxylation and oxygenation reactions make it an important target to increase plant photosynthesis and yield (Evans, 2013). In C3 plants, the Rubisco comprises as high as 50% of total leaf protein and the high concentration makes it better to balance the photorespiration processes (Woodrow and Berry, 1988; Quick et al., 1991). The activity of Rubisco and transcription of the small subunit, which is diverse in the different forms is obviously increased when exposed to the environment of the greenhouse with RPO film coverage, making it possible to demonstrate higher Pn (Figure 3). All of these may be the potential to promote the fruit yield.

Recently, rare-earth conversion film has been focused on to adjust the light quality and improve the light transmittance in a solar greenhouse. In this experiment, the projection ratios of ultraviolet, violet, and green light are reduced and the projection ratios of blue, red-orange, and far-red light are induced in the solar greenhouse covered by RPO film. Different light conversion agents added into the film may cause different light spectrum (Kang et al., 2022). Additionally, the study found that rare-earth conversion film improved light transmittance, consistent with the previous research (Liu et al., 2022). Rare-earth light conversion films can improve the greenhouse environment significantly. The present study found that the light intensity, air temperature, and soil temperature (10 cm soil layer) were higher in the RPO greenhouse than that in the PO film greenhouse (Figure 1), which is in accordance with others (Garofalakis et al., 2006; Xin et al., 2019; Yu et al., 2019). That may be the reason for the enhanced photosynthesis, growth and yield (Figure 3; Table 4; Supplementary Tables S3, S4; Trouwborst et al., 2010; Kong et al., 2021).

In plants, mesophyll is a major tissue for photosynthesis, and the mesophyll cells are the major RNA source, which provide as high as 80% of total RNA (Uemoto et al., 2018). There are two sub-tissue types in mesophyll tissue, the palisade and spongy mesophyll, whose functions are different markedly. Palisade cells are packed tightly together and have many chloroplasts. Most of the plant’s photosynthesis is carried out in this sub-tissue. Whereas the spongy cells have many air space which helps spongy cells to assimilate CO2. Thus the spongy tissue mainly participates in breath (Uemoto et al., 2018). In our study, changes in the light quality effects leaf structure (Dieleman et al., 2019). Blue light has been shown to increase the thickness of leaf epidermis and palisade tissue and change the tightness of palisade tissue and sponge tissue (Sæbø et al., 1995; Macedo et al., 2011). In the greenhouse with RPO film, longer and more tightly arranged palisade cells can help to accumulate more chloroplasts. Light quality can also adjust the number of stomata in leaves. Studies have demonstrated that far-red light-absorbing phytochromes (phys) also play a role in the control of stomatal aperture (Chen et al., 2012). Similarly, improved number of stomata in sweet pepper leaves was observed in this experiment (Table 2). Leaf thickness and stomatal length of RPO treatment increased by 21.43 and 26.70%, respectively (Table 2), compared with those of CK. It may be the reason for the increased net photosynthetic rate and yield. This study can play a positive role in exploring measures for economically efficient utilization of facility cultivation.

## Conclusion

In this study, we found that covering a greenhouse with a rare-earth light conversion film converted the ultraviolet light, purple light, and green light to blue, red, orange, and far-red light and improved the relative light intensity, air temperature, and soil temperature. These changes improved growth and yield by advancing photosynthesis, and improved fruit quality of sweet pepper through adjusting endogenous hormone content in the low-temperature seasons. Thus, the findings suggest that using RPO will effectively increase productivity and improve the fruit quality of sweet pepper in a greenhouse.

## Data availability statement

The raw data supporting the conclusions of this article will be made available by the authors, without undue reservation.

## Author contributions

QL designed the experiments. YG and GL performed the experiments. QL and YG wrote the manuscript and analyzed the data. BC, ZZ, NL, and YL edit and revised the manuscript. All authors contributed to the article and approved the submitted version.

## Funding

This work was supported by China Agriculture Research System of MOF and MARA (grant number CARS-24-G-03), earmarked fund for Modern Agro-industrial Technology Research System of Hebei (grant number HBCT2021030214).

## Conflict of interest

The authors declare that the research was conducted in the absence of any commercial or financial relationships that could be construed as a potential conflict of interest.

## Publisher’s note

All claims expressed in this article are solely those of the authors and do not necessarily represent those of their affiliated organizations, or those of the publisher, the editors and the reviewers. Any product that may be evaluated in this article, or claim that may be made by its manufacturer, is not guaranteed or endorsed by the publisher.

## Abbreviations

ABA: Abscisic acid
GA3: Gibberellic acid 3
IAA: Indole-3-acetic acid
LEDs: Light-emitting diodes
PFD: Light quantum flux density
Pn: Net photosynthetic rate
PO: Polyolefin film
qRT-PCR: Quantitative real-time PCR
RPO: Rare-earth light conversion film
TTC: 2, 3, 5-Tripheyl-2H-tetrazolium chloride
ZR: Zeatin Riboside
