# Supplementary material for: Effects of rare-earth light conversion film on the growth and fruit quality of sweet pepper in a solar greenhouse
Source: Front Plant Sci. 2022 Sep 6;13:989271. doi: 10.3389/fpls.2022.989271 (PMC9485565; doi:10.3389/fpls.2022.989271)
Supplement: Supplementary file 1 [file Data_Sheet_1.docx]

**Supplementary materials**

Table S1 qRT-PCR primer sequences

| Primer names | Sequences (5’-3’) |
| --- | --- |
| RbcL-F | GAGTTGTAGGGAGGGATT |
| RbcL-R | TCGGTCCATACAGTTATC |
| RbcS-F | CAATGCATGCTGGTATGGCC |
| RbcS-R | GGGTAGCATCAGTACACCCG |
| Ubi3-F | TGTCCATCTGCTCTCTGTTG |
| Ubi3-R | CACCCCAAGCACAATAAGAC |

Table S2 Effect of RPO film on photosynthetic pigment content of sweet pepper leaves

| Treatment | Chlorophyll a  (mg g^-1^ FW) | Chlorophyll b  (mg g^-1^ FW) | Carotenoids  (mg g^-1^ FW) |
| --- | --- | --- | --- |
| CK | 15.21±0.33b | 3.65±0.30b | 3.81±0.39a |
| RPO | 17.91±0.30a | 4.69±0.28a | 4.36±0.52a |

Note: All values are presented as the mean ± SD (n = 5). Lowercase letters indicate that the mean values are significantly different among samples (p < 0.05).

Table S3 Effects of RPO film on the plant growth of sweet pepper in the greenhouse.

| Treatment | Plant height (cm) | Stem diameters (mm) | Leaf length (cm) | Leaf width (cm) | Internode length (cm) |
| --- | --- | --- | --- | --- | --- |
| CK | 75.38±3.47b | 11.84±0.50b | 22.40±1.10a | 11.40±0.99a | 4.63±0.58b |
| RPO | 83.71±2.14a | 13.85±0.87a | 22.78±1.09a | 11.88±0.77a | 5.80±0.54a |

Note: All values are presented as the mean ± SD (n = 5). Lowercase letters indicate that the mean values are significantly different among samples (p < 0.05).

Table S4 Effect of RPO film on the biomass of sweet pepper plants in the greenhouse.

| Treatment | Fresh weight | | |  | Dry weight | | |
| --- | --- | --- | --- | --- | --- | --- | --- |
|  | root (g plant^-1^) | Stem (g plant^-1^) | Leaf (g plant^-1^) |  | Root (g plant^-1^) | Stem (g plant^-1^) | Leaf (g plant^-1^) |
| CK | 29.29±0.62b | 211.60±11.61b | 221.33±9.87b |  | 7.13±0.11b | 30.86±1.14b | 35.16±2.85b |
| RPO | 40.08±0.89a | 314.44±14.48a | 281.12±9.82a |  | 9.72±0.11a | 49.86±1.26a | 47.12±2.30a |

Note: All values are presented as the mean ± SD (n = 5). Lowercase letters indicate that the mean values are significantly different among samples (p < 0.05).

Table S5 Correlation matrix of sweet pepper yield and quality indicators

| Treatment | Vc | Soluble protein | Organic acid | Soluble sugars | Free amino acids | Fruit length | Fruit width | Average weight per fruit | Yield |
| --- | --- | --- | --- | --- | --- | --- | --- | --- | --- |
| Vc | 1 |  |  |  |  |  |  |  |  |
| Soluble protein | 0.59 | 1 |  |  |  |  |  |  |  |
| Organic acid | -0.379 | -0.831** | 1 |  |  |  |  |  |  |
| Soluble sugars | 0.667* | 0.983** | -0.839** | 1 |  |  |  |  |  |
| Free amino acids | 0.402 | 0.153 | 0.274 | 0.157 | 1 |  |  |  |  |
| Fruit length | 0.337 | 0.743* | -0.607 | 0.670* | -0.066 | 1 |  |  |  |
| Fruit width | 0.483 | 0.48 | -0.337 | 0.564 | 0.03 | 0.074 | 1 |  |  |
| Average weight per fruit | 0.671* | 0.974** | -0.820** | 0.990** | 0.184 | 0.625 | 0.536 | 1 |  |
| Yield | 0.645* | 0.976** | -0.851** | 0.997** | 0.154 | .644* | 0.55 | 0.993** | 1 |

*Correlation is significant at the 0.05 level (two-tailed).

**Correlation is significant at the 0.01 level (two-tailed)


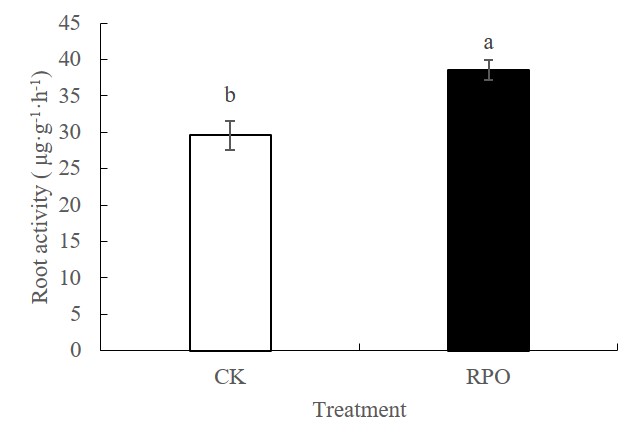


Fig. S1 Effect of rare earth light-conversion film on root activity of sweet pepper plants. All values are presented as the mean ± SD (n = 5). Lowercase letters indicate that the mean values are significantly different among samples (p < 0.05).
